# Supplementary figures and images for: Predictive Factors for Bilateral Disease in Papillary Microcarcinoma: A Retrospective Cohort Study
Source: Curr Oncol. 2022 Aug 23;29(9):6010–7. doi: 10.3390/curroncol29090473 (PMC9497734; doi:10.3390/curroncol29090473)

**Supplement Figure S1. Overall collective.**

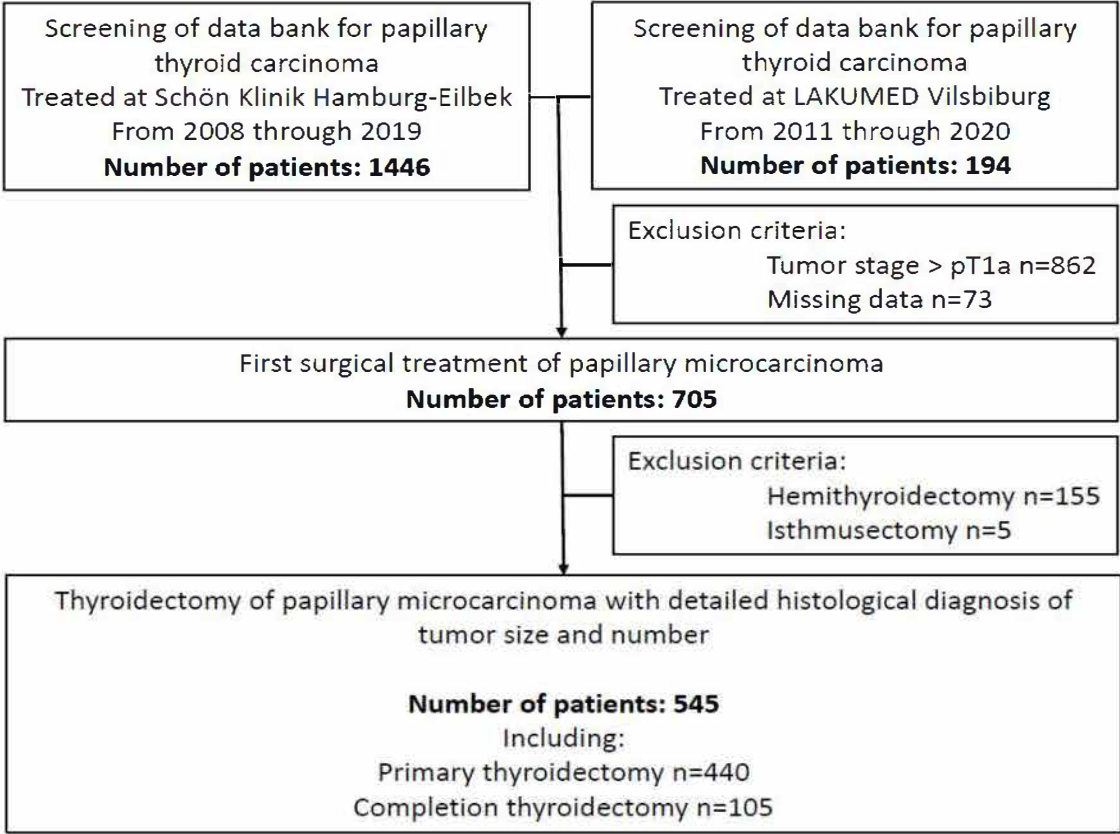

Supplement: Supplementary file 1 [file curroncol-29-00473-s001.zip › curroncol-1821503-supplementary.pdf]
